# Benign Histopathologic Findings of Endometrium among Perimenopausal Women presenting with Abnormal Uterine Bleeding: A Descriptive Cross-sectional Study

**DOI:** 10.31729/jnma.7146

**Published:** 2021-11-30

**Authors:** Ramesh Dhakhwa, Rashmi Bhattarai, Jayanti Shah, Archana Shakya, Sailesh Pradhan

**Affiliations:** 1 Department of Pathology, Kathmandu Medical College, Sinamangal, Kathmandu, Nepal; 2 Department of Gynecology and Obstetrics, Paropakar Maternity and Women's Hospital, Kathmandu, Nepal

**Keywords:** *abnormal uterine bleeding*, *histopathology*, *perimenopausal*

## Abstract

**Introduction:**

Abnormal uterine bleeding is the most common presenting complaint in the perimenopausal age group. Endometrial biopsy obtained by dilatation and curettage is the preferred modality of investigation to determine the causative pathology of abnormal uterine bleeding. The objective of this study was to find out the prevalence of the benign histopathological findings in perimenopausal women presenting with abnormal uterine bleeding.

**Methods:**

This descriptive cross-sectional study was conducted among patients between 1st June 2020 to 30th September 2021. Ethical approval was taken from the Institutional Review Committee of Kathmandu Medical College (reference number: 305202002). Using the convenience sampling method, 96 cases of endometrial biopsies were studied under light microscopy. Data was analyzed using the Statistical Package for the Social Sciences version23.0. Point estimate at 95% Confidence Interval was calculated along with frequency and proportion for binary data.

**Results:**

Among the 96 specimens, the prevalence of benign findings was 93 (96.9%) (93-100 at 95% Confidence Interval). Among them, the commonest benign histopathologic spectrum was hormonal imbalance pattern in 40 (41.7%) followed by normal menstrual pattern 35 (36.5%). Five (5.2%) cases showed chronic endometritis. Six (6.2%) cases of endometrial hyperplasia without atypia were identified. Three (3.1%) cases showed endometrial atrophy. Four (4.1%) cases showed endometrial polyp.

**Conclusions:**

The prevalence of benign histopathological findings among endometrial biopsies in the study was similar to other studies.

## INTRODUCTION

Abnormal uterine bleeding (AUB) is any type of menstrual bleeding that does not fall within the normal ranges for amount, frequency, duration or cyclicity.^1^ It is the most common presenting complaint in gynecology outpatient department, especially in perimenopausal age group.^2^ Perimenopause is transitional years prior to menopause which may be due to variation in normal cyclical pattern as a result of physiological hormonal changes.AUB with abnormal blood loss has a profound effect on the quality of the woman's life.

However, most of the cases of AUB in perimenopausal age are of benign nature without requiring any invasive surgeries. Endometrial biopsy obtained by dilatation and curettage is the preferred modality of investigation to determine the causative pathology of AUB.^3^ Histopathologic changes on endometrium may vary from physiological findings to overt pathologic features in patients with AUB in different population and age group. So far limited data is available regarding the endometrial patterns seen in perimenopausal women in our population.

The objectives of this study were to find out the prevalence of benign histopathological findings in perimenopausal women presenting with AUB.

## METHODS

This was a descriptive cross-sectional study conducted among patients 1st June 2020 to 30^th^ September 2021. The institutional review committee of Kathmandu Medical College and Teaching Hospital provided the ethical approval (reference number:305202002). Using convenience sampling technique, endometrial biopsy specimen submitted to the Department of Pathology for histopathologic evaluation during the study period were included in the study. The sample size was calculated as,

n = Z^2^ × p × q / e^2^

  = (1.96)^2^ × (0.5) × (1-0.5) / (0.1)^2^

  = 96

where,

n= required sample size,
Z= 1.96 at 95% Confidence Interval (CI),
p= prevalence taken as 50% for maximum sample size,
q= 1-p
e= margin of error, 10%

Therefore, the calculated sample size was 96. Hence, 96 endometrial samples were collected. The endometrial samples were fixed in 10%formalin for 12-24 hours and the entire tissue was submitted for routine processing. 5|j thickness sections taken from paraffin blocks were stained with Haematoxylin and Eosin (H&E) and studied under light microscopy by a pathologist. Patients with cervical-vaginal pathology and systemic causes of abnormal uterine bleeding were excluded from the study. Relevant demographic data were obtained from requisition form provided with the specimen.

Data were entered and analyzed using the Statistical Package for the Social Sciences version 23. Point estimate at 95% Cl was calculated along with frequency and proportion for binary data.

## RESULTS

In the present study, out of the 96 cases of endometrial biopsies included, the prevalence of benign histopathological findings was 93 (96.9%) (93-100 at 95% CI). Only three (3.1%) cases showed histopathologic evidence of endometrial malignancy. Majority of patients with AUB had a hormone imbalance pattern on endometrial biopsy 40 (41.7%). Endometrial atrophy was noted in three (3.1%) cases. Six (6.2%) cases showed endometrial hyperplasia without atypia. Five (5.2%) cases showed features of endometritis. In four (4.1%) cases AUB was due to endometrial polyp (Table 1).

**Table 1:** Benign Histopathologic findings in endometrial biopsy (n= 96).

S.N.	Diagnosis	n (%)
1.	Hormonal imbalance pattern (DUB, stromal and glandular breakdown, pill effect)	40 (41.7)
2.	Proliferative phase endometrium	24 (25)
3.	Secretory phase endometrium	11 (11.5)
4.	Endometrial hyperplasia without atypia	6 (6.2)
5.	Chronic endometritis	5 (5.2)
6.	Endometrial polyp	4 (4.1)
7.	Atrophy	3 (3.1)
	Total	93 (96.9)

Histopathologic changes favoring hormone imbalance included disordered proliferative endometrium 32 (80%), non-secretory endometrium with endometrial and stromal breakdown in 3 (7.5%) and pill effect in 5 (12.5%) cases.

**Figure 1:**
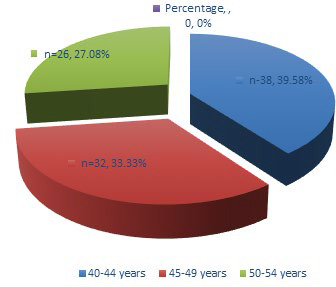
Agewise distribution of AUB.

Most of the women presenting with AUB fell into the 40-44-year age group, 38 (39.58%), followed by 45-49 year age group, 32 (33.33%) and 50-54 year age group, 26 (27.08%) (Figure 1).

Menorrhagia, in 35 (36.4%) cases, was the most common presenting symptom (Table 2).

**Table 2:** Clinical pattern of AUB.

S.N.	Bleeding Pattern	n (%)
1.	Menorrhagia	35 (36.4)
2.	Polymenorrhoea	25 (26)
3.	Menometrorrhagia	17 (17.7)
4.	Intermenstrual bleeding	12 (12.5)
5.	Metrorrhagia	5 (5.2)
6.	Oligomenorrhea	2 (2)
	Total	96 (100)

Despite having AUB, a significant number of cases showed normal cyclical endometrium where 24 (25%) showed proliferative endometrium and 11 (11.5%) showed secretory endometrium.

## DISCUSSION

The endometrium undergoes a complex regular cycle of periodic proliferation, differentiation, breakdown and regeneration which forms the basis of normal menstrual cycle; any deviation from it would result in abnormal uterine bleeding. Clinically AUB manifests as menorrhagia, polymenorrhea, polymenorrhagia, metrorrhagia, menometrorrhagia, intermenstrual bleeding, etc.^4,5^ A new classification for the causes of abnormal uterine bleeding is based on the acronym PALM-COEIN - Polyps, Adenomyosis, Leiomyoma, Malignancy, Hyperplasia, Coagulopathy, Ovulatory disorders, Endometrial causes, Iatrogenic and Not classified, was developed by the International Federation of Gynaecology and Obstetrics in November 2010.^6,7,8^ When unscheduled bleeding occurs, especially if it is heavy or prolonged, further investigations such as trans-vaginal ultrasound and, hysteroscopy with biopsy if indicated, are needed.^9^ The accuracy of endometrial biopsy for the detection of endometrial abnormalities has been reported to be as high as 96%.^10^ Diagnosis and the management strategies of AUB is not complete without evaluation of histopathological characteristics of endometrial biopsy.^11^ Endometrial biopsy tissue obtained by a simple procedure of D and C (Dilation and curettage) is an effective and safe diagnostic step in evaluation of AUB and for the diagnosis of endometrial pathologies.^12,13^

AUB is common in the perimenopausal age group. In a study conducted by Chapagain in a tertiary hospital in Nepal, majority of cases of AUB seen in perimenopausal age group was between 40-44 years (45.5%).^14^ We also observed that the common age of AUB was noted in the 40-44 years age group (39.58%) followed by 45-49 years age group (33.33%) and 50-54 years age group (27.08%) which is similar to the study done by Valson and Bhatiayani, et al. Probable reason of increased incidence of AUB in this age group (40-50 years) is that the women in this age group are in their climacteric period. As women approach menopause, cycles shorten and become intermittently anovulatory due to the decline in the level of ovarian follicles and estradiol level.^15,16^

Menorrhagia (36.4%) was the most common presentation followed by polymenorrhea (26%) in our study. In the study conducted by Chapagain, menorrhagia was the commonest presenting complaint (40.3%) followed by menometrorrhagia (23.4%).^14^ which was similar to the observation made by Chaudhary, Razzaq, et al. and Shrestha in their studies which were conducted in a population of similar race, socio-economic status and environment as our study.^17,18,19^ Study conducted by Abid, et al. however found polymenorrhea as the commonest presenting complaint.^20^

Various studies have shown benign histopatholgical changes in patients presenting with AUB.^14-22^ In our study the commonest histological pattern in perimenopausal women was hormonal imbalance pattern (41.7%) followed by normal cyclical pattern (proliferative and secretory pattern combined, 36.5%). Among the cases showing hormone imbalance patterns, histomorphologic features showed predominantly disordered proliferative endometrium (32/40 cases), glandular and stromal breakdown (3/40 cases) and pill effect (5/40 cases). Abid, et al. also reported hormonal imbalance pattern was the commonest in perimenopausal age group (54%). This correlates with the fact that perimenopausal age is transition from normal ovulation to anovulation.^20^

Thirty-five women with AUB showed normal cyclical endometrium which was the second common pattern in our study. Out of these 36 cases, 24 (25%) showed proliferative endometrium and 11 (11.5%) revealed secretory phase endometrium. This is in contrast to the studies done by Das et al, Razzaq et al, Bhatiyani and Singh, et al. who reported normal cyclical pattern to be the commonest pattern of endometrium. Their studies included endometrial biopsy in a wide age range while our study took into account only cases which fell into the perimenopausal age group (40-54 years).^16,18,21,22^

Endometrial pathologies such as endometrial hyperplasia, polyps, submucous leiomyoma and endometrial carcinoma should be suspected and evaluation of endometrium is necessary in AUB in perimenopausal women.^23^ We observed endometrial hyperplasia without atypia in six cases (6.2%). Three of these cases were present in the 45-49 year age group and the other three in the 50-54 years age group. Endometrial hyperplasia without atypia is a proliferation of endometrial glands of irregular size and shape without significant cytological atypia. It is most commonly diagnosed in perimenopause with symptoms of abnormal, non-cyclical vaginal bleeding. It is a result of prolonged estrogen exposure unopposed by progesterone or progestational agents acting on the entire endometrial field.^24^ In the study done by Abid et al, endometrial hyperplasia was present in 5% of cases withAUB and the incidence was most common in the postmenopausal and perimenopausal age group.^20^

We observed inflammatory pathology only in 5 cases (5.2%). All these cases showed features of non-specific chronic endometritis. Abid, et al. reported a lower incidence of endometritis (9.1%) in perimenopausal age group than in reproductive age group (18%) which they attributed to the fact that the women in reproductive age group had a greater chance of exposure to casesarian sections, spontaneous and therapeutic abortion and intrauterine contraceptive device etc. hence prone to develop chronic endometritis. Perveen, et al. found chronic endometritis in a larger number of cases (37%).^25^ This variation may be due to socioeconomic status, hygienic conditions or exposure to surgical intervention.

Endometrial polyp was present in four cases (4.1%). None of the polyps showed atypical hyperplasia or carcinoma. Azim, et al. demonstrated an increasing frequency of polyp with advancing age.^26^A small number of cases demonstrated atrophic endometrium (3.1%). All three cases were in the 50-54 age group and no history of menopause was present in them. This pattern is most common in post-menopausal women however, anovulation seen in perimenopasual age group eventually leads to permanent loss of ovarian function manifested as atrophic or inactive endometrium.^20^

Our study was a descriptive hospital-based study carried in a selected age group of women in a short duration of time, therefore might not be representative of the entire population. However,this study would provide a database with regards to benign histopathological patterns in AUB in perimenopausal age groups.

## CONCLUSIONS

The prevalence of benign histopathological findings among endometrial biopsies in the study was similar to other studies. Hormonal imbalance pattern is the predominant benign histopathologic pattern seen in perimenopausal women presenting with AUB. Histopathologic findings in an endometrial biopsy helps gynaecologists to decide appropriate management strategies.
